# Intermittent Hypoxia Induces Autophagy to Protect Cardiomyocytes From Endoplasmic Reticulum Stress and Apoptosis

**DOI:** 10.3389/fphys.2019.00995

**Published:** 2019-08-07

**Authors:** Jui-Chih Chang, Wei-Fen Hu, Wen-Sen Lee, Jian-Hong Lin, Pei-Ching Ting, Huai-Ren Chang, Kun-Ruey Shieh, Tsung-I Chen, Kun-Ta Yang

**Affiliations:** ^1^Department of Surgery, Hualien Tzu Chi Hospital, Buddhist Tzu Chi Medical Foundation, Hualien, Taiwan; ^2^School of Medicine, Tzu Chi University, Hualien, Taiwan; ^3^Master Program in Medical Physiology, School of Medicine, Tzu Chi University, Hualien, Taiwan; ^4^Graduate Institute of Medical Sciences, School of Medicine, College of Medicine, Taipei Medical University, Taipei, Taiwan; ^5^PhD Program in Pharmacology and Toxicology, School of Medicine, Tzu Chi University, Hualien, Taiwan; ^6^Division of Cardiology, Department of Internal Medicine, Hualien Tzu Chi Hospital, Buddhist Tzu Chi Medical Foundation, Hualien, Taiwan; ^7^Department of Physiology, School of Medicine, Tzu Chi University, Hualien, Taiwan; ^8^Center for Physical Education, College of Education and Communication, Tzu Chi University, Hualien, Taiwan; ^9^Institute of Education, College of Education and Communication, Tzu Chi University, Hualien, Taiwan

**Keywords:** apoptosis, autophagy, cell death, endoplasmic reticulum, intermittent hypoxia, stress

## Abstract

Intermittent hypoxia (IH), characterized as cyclic episodes of short-period hypoxia followed by normoxia, occurs in many physiological and pathophysiological conditions such as pregnancy, athlete, obstructive sleep apnea, and asthma. Hypoxia can induce autophagy, which is activated in response to protein aggregates, in the proteotoxic forms of cardiac diseases. Previous studies suggested that autophagy can protect cells by avoiding accumulation of misfolded proteins, which can be generated in response to ischemia/reperfusion (I/R) injury. The objective of the present study was to determine whether IH-induced autophagy can attenuate endoplasmic reticulum (ER) stress and cell death. In this study, H9c2 cell line, rat primary cultured cardiomyocytes, and C57BL/6 male mice underwent IH with an oscillating O_2_ concentration between 4 and 20% every 30 min for 1–4 days in an incubator. The levels of LC3, an autophagy indicator protein and CHOP and GRP78 (ER stress-related proteins) were measured by Western blotting analyses. Our data demonstrated that the autophagy-related proteins were upregulated in days 1–3, while the ER stress-related proteins were downregulated on the second day after IH. Treatment with H_2_O_2_ (100 μM) for 24 h caused ER stress and increased the level of ER stress-related proteins, and these effects were abolished by pre-treatment with IH condition. In response to the autophagy inhibitor, the level of ER stress-related proteins was upregulated again. Taken together, our data suggested that IH could increase myocardial autophagy as an adaptive response to prevent the ER stress and apoptosis.

## Introduction

The endoplasmic reticulum (ER), which possesses the structural and functional features of an organelle, supports the regulation of synthesis, folding, and orderly transport of proteins (Gorlach et al., 2006). Disruption of ER functions can induce an accumulation of unfolded and misfolded proteins in the ER lumen, a condition known as ER stress. Acute or mild ER stress can promote cell survival and adaptation (Oslowski and Urano, 2011). In contrast, chronic or prolonged ER stress can trigger apoptosis (Toth et al., 2007). In stress situations, cells initiate unfolded protein responses (UPRs) including protein kinase RNA-like ER kinase (PERK), inositol-requiring protein 1 (IRE1), and activating transcription factor 6 (ATF6), three major proteins on the ER membrane to alleviate the disturbance of ER homeostasis (Ivanova and Orekhov, 2016).

ER stress has been implicated in the pathogenesis of several diseases such as neurodegeneration, brain I/R, diabetes (Toth et al., 2007), and cardiovascular diseases such as heart failure, ischemic heart disease, cardiac hypertrophy, and atherosclerosis (Minamino and Kitakaze, 2010). Exposure of hypoxia/reoxygenation, also called intermittent hypoxia (IH), has been linked to ER stress (Cai et al., 2014; Xu et al., 2015). For instance, ER has been indicated to play a major role in adaptive responses to I/R-caused injury. The change of IH in obstructive sleep apnea hypopnea syndrome is similar to that of I/R (Cai et al., 2014). Moreover, it has been demonstrated that chronic IH could activate ER stress, subsequently causing cardiomyocytic apoptosis (Xu et al., 2015). However, roles of ER responses in mediating pathophysiological reactions in IH are still not well known.

IH commonly occurs in many physiological conditions, such as vigorous exercise or high-altitude sojourns, and pathophysiological conditions, such as chronic obstructive pulmonary disease and obstructive sleep apnea (Kopelman et al., 1986; Foster et al., 2007). IH can lead to an increase of intracellular reactive oxygen species (ROS) generation (Chen et al., 2014). It has been reported that unfolded proteins’ accumulation in the ER causes Ca^2+^ leakage into the cytosol *via* inositol trisphosphate receptor (IP3R), subsequently evoking influx of Ca^2+^ into the nuclei and the mitochondria and resulting in the generation of ROS (Mahdi et al., 2016). However, ROS generation in cardiomyocytes induced by IH promotes Ca^2+^ efflux from the cytosol to the extracellular milieu and Ca^2+^-mediated ER regulation (Chen et al., 2014). In addition, IH-induced physiological and pathological responses exert beneficial effects on protecting cardiovascular and respiratory system against ischemia injury (Powell and Garcia, 2000; Fletcher, 2001; Neubauer, 2001) and preventing arrhythmia (Meerson et al., 1987; Zhang et al., 2000). The IH-mediated autophagy is able to maintain contractile function by attenuating myocytes necrosis (Maeda et al., 2013; Gui et al., 2016).

Autophagy plays an important role in cellular homeostasis and is the process by which cells recycle cytoplasm and dispose of excess or damaged organelles (Hamacher-Brady et al., 2007). In most tissues, the basal level of autophagy is low and involved in regulating the routine cytoplasmic components turnover. However, autophagy can be increased by environmental changes, such as IH and nutrient depletion (Ogata et al., 2006), and in some heart diseases, hence promoting survival. However, large amounts of autophagy may be maladaptive (Maeda et al., 2013). In mammalian systems, autophagy has been shown to be involved in many physiological processes, such as antiaging mechanisms, cell growth control, the response to starvation, and innate immunity. On the other hand, the deregulation of autophagy has been suggested to play important roles in some diseases, such as cardiomyopathy, cancer, neurodegenerative disorders, and muscular diseases (Levine and Klionsky, 2004).

Notably, it has been indicated that chronic IH exposure and acute IH exposure have different effects on ER stress. Chronic IH leads to an increase in ER stress, while acute IH does not (Sforza and Roche, 2016). This is probably because acute IH may upregulate the autophagy level to alleviate ER stress. However, prolonged or severe ER stress can cause cell death (Collett et al., 2018). Until now, whether autophagy is involved in the acute IH-attenuated ER stress has still been unclear. Moreover, acute IH can exert several preconditioning effects, including activation of anti-apoptosis proteins, and improving Ca^2+^ handling against myocardial infarction (Lien et al., 2018). Therefore, the aim of the present study was to examine whether the IH-induced autophagy can protect cells against the ER stress-induced apoptosis.

## Materials and Methods

### Chemicals and Solutions

Ethylenediaminetetraacetic acid (EDTA), fetal bovine serum (FBS), penicillin, and trypsin were from Gibco/Life Technologies (Rockville, MD, USA). All other chemicals, such as 3-(4,5-dimethylthiazol-2-yl)-2,5-diphenyltetrazolium bromide (MTT), dimethyl sulfoxide (DMSO), thapsigargin (TG), curcumin (Cur), H_2_O_2_, 3-methyladenine (3MA), chloroquine (CQ), and wortmannin (Wort) were purchased from Sigma (Saint Louis, MO, USA). LC3, p-eIF2, CHOP and cleaved PARP antibodies were from Cell signaling (Danvers, MA, USA). GRP78/Bip, ATF4 and p-IRE1 antibodies were purchased from Abcom (Cambridge, MA, USA). ATF6 antibody was ordered from Proteintect (Rosemont, IL, USA). GAPDH antibody was from Santa Cruz (CA, USA).

### Cells

It has been demonstrated that H9c2 cells are more similar to primary cultured cardiomyocytes than the atrial HL-1 cells. H9c2 cells can be successfully used to simulate cardiac hypoxia-reoxygenation injury (Kuznetsov et al., 2015). An adherent H9c2 cell line of rat embryonic cardiomyocytes (ATCC, Manassas, VA, USA) was used for the *in vitro* study. Cells were grown in Dulbecco’s Modified Eagle Medium-High Glucose (DMEM-HG, Thermo, Cleveland, OH, USA) supplemented with 10% FBS, 3.7 g/L sodium bicarbonate, and 100 units/ml penicillin in a humidified atmosphere of 5% CO_2_ at 37°C. The medium was replaced every other day.

### Rat Primary Cultured Cardiomyocytes

Two-day-old Sprague-Dawley rats (males and females) were sacrificed by cervical dislocation and decapitated. The cardiomyocytes were dissociated from the ventricles through repeated digestion in enzyme solution (0.051% pancreatin and 0.01% collagenase in Hank’s solution), followed by enzyme inactivation using an F-12 medium [80% F-12 nutrient mixture, 10% fetal bovine serum (FBS), 10% horse serum, and 1% penicillin]. Cells were plated on a 10-cm dish and incubated for 1 h at 37°C in a 5% CO_2_ incubator to remove fibroblasts. Ventricular myocytes were plated on 35-mm collagen-coated dishes and grown in F-12 medium with 10 μM cytosine arabinoside, and the medium was daily replaced. All procedures were performed in accordance with the Animal Care Guidelines of the Tzu Chi University.

### Animals

C57BL/6 male mice (12–14 weeks old) purchased from BioLASCO Taiwan Co. Ltd. were maintained in an artificial 12-h light-dark cycle and given free access to water and food in the Laboratory Animal Center, Tzu Chi University. All experimental and surgical procedures were performed following the recommended procedures approved by the Institutional Animal Care and Use Committee of Tzu Chi University (PPL number: 107104), and conform to the guidelines from Directive 2010/63/EU of the European Parliament on the protection of animals used for scientific purposes or the NIH guidelines. All mice were sacrificed by cervical dislocation. The proceeding for heart isolation was previously described (Chen et al., 2015).

### Intermittent Hypoxia in Cells

After seeding, cells were allowed to grow for 1 day in an incubator with an hypoxia incubator subchamber system, and then the hypoxia incubator subchambers were filled with normoxia (20% O_2_, 5% CO_2_, and 75% N_2_) or IH (4% O_2_ to peak 20% O_2_ with 5% CO_2_, and 75% N_2_ to peak 91% N_2_, 1 cycle/h) for 24 h/day followed by drug treatment. The medium with or without drugs was changed daily during IH processes. In addition, a previous study suggested that 3 days of IH can induce non-lethal ROS levels that enhance the Ca^2+^-handling ability of cardiomyocytes (Chen et al., 2014). Therefore, 3 days of IH was adopted to investigate the effects of IH on ER stress.

### Intermittent Hypoxia in Animals

C57BL/6 male mice underwent room air (RA) or IH (4% O_2_ to peak 20% O_2_ with 5% CO_2_ at a rate of 75 s/cycle) in the same plexiglass cylindrical chambers with snug-fitting lids placed next to the cage equipped with the IH apparatus for 8 h/day during the “light on” period of 12 h. For drug treatment, mice were intraperitoneally (i.p.) injected with indicated drugs prior to IH. After treatment, the mouse was cervically dislocated. The mouse heart was harvested, and then stored at −80°C until analyses. The animal number used in each group is described in figure legends.

### Western Blotting

H9c2 cells (1 × 10^5^ cells/ml) or heart ventricle tissues (30 mg) were lysed by sonication on ice with 100-μl RIPA lysis buffer (cat. no. 20-188; Millipore, USA) containing 1% protease inhibitor for H9c2 cells and 200-μl RIPA lysis buffer containing 1% phosphatase inhibitor for heart, respectively, and then centrifuged at 12,000 rpm at 4°C for 10 min. Proteins (30 μg/lane) resolved on 10% sodium dodecyl sulfate-polyacrylamide gel using the Bis-Tris Electrophoresis System (Bio-Ray, USA) were transferred to polyvinylidene fluoride membranes (Bio-Ray, USA), blocked with non-fat milk or 5% BSA at RT for 1 h or 4°C overnight, and then incubated with primary antibodies against LC3 (1:2,000), p-eIF2 (1:2,000), CHOP (1:1,000), cleaved PARP (1:1,000), GRP78/Bip (1:5,000), ATF4 (1:2,000), p-IRE1 (1:2,000), cleaved ATF6 (1:2,000), or GAPDH (1:10,000) at 4°C overnight. After incubation, the membranes were treated with the horseradish peroxidase-labeled secondary antibody at RT for 1 h, and then washed with PBS. Protein levels were measured using RPN2232 ECL™ Prime Western Blotting Detection Reagent (GE Healthcare, Piscataway, NJ, USA) and X-ray films (GE Healthcare, USA) or UVP BioSpectrum. The resulting bands were captured with a scanner and quantified as arbitrary units (OD × band area) using the Image J software (National Institutes of Health, Bethesda, MD, USA).

### Autophagic Flux Analysis

Autophagy flux was analyzed by confocal microscopy (Nikon C2 Si+) using DQ™-Green BSA (self-quenched green BODIPY dye conjugated to BSA; Molecular Probes). H9c2 cells were placed on 12-mm coverslips; exposed under IH for 72 h; incubated with DQ™-Green BSA (10 μg/ml) in DMEM under IH at 37°C for additional 24 h; washed twice with N-2-hydroxy-ethylpiperazine-N′-2-ethanesulphonic acid (HEPES)-buffered Tyrode solution (NT) composed of 10 mM HEPES, 11 mM glucose, 140 mM NaCl, 4.5 mM KCl, 2.0 mM CaCl_2_, 1.2 mM KH_2_PO_4_, and 1.0 mM MgCl_2_ with pH adjusted to 7.4 with NaOH at 37°C buffer to remove excess probe; fixed with 10% formaldehyde at RT for 1 h; and then permeabilized with 0.15% Triton X-100 at RT for 15 min. To block the nonspecific binding sites, cells were treated with 5% dried non-fat milk in NT buffer at RT for 15 min. After blocking, cells were incubated with anti-LC3 antibody (Cell Signaling, MA, USA) at a 1:200 dilution overnight at 4°C, washed twice with NT buffer, and then incubated with rabbit IgG antibody (Dylight 594, Gene Tex, San Antonio, TX, USA) at a 1:200 dilution.

### Terminal-Deoxynucleoitidyl Transferase Mediated Nick End Labeling Assay

The apoptotic cells were detected with Terminal-deoxynucleoitidyl Transferase Mediated Nick End Labeling (TUNEL) staining according to the manufacturer’s instructions (*In Situ* Cell Death Detection Kit, Fluorescein, Roche). Images were captured by confocal microscopy (Nikon C2 Si+; Melville, NY. USA). Apoptosis was quantified by determining the percentage of positively stained cells for all of the nuclei (1 μM Hoechst33342) in three randomly chosen fields at 40× magnification.

### Statistics

Values represent the mean ± SEM from at least three independent experiments. Comparisons between two groups were subjected to Student’s *t* test. Comparisons between more than two groups were subjected to one-way ANOVA test followed by Tukey’s *post hoc* test. Significance was accepted at *p* < 0.05.

## Results

### Effects of Intermittent Hypoxia on Endoplasmic Reticulum Stress in Cultured H9c2 Cells

**Figure 1:**
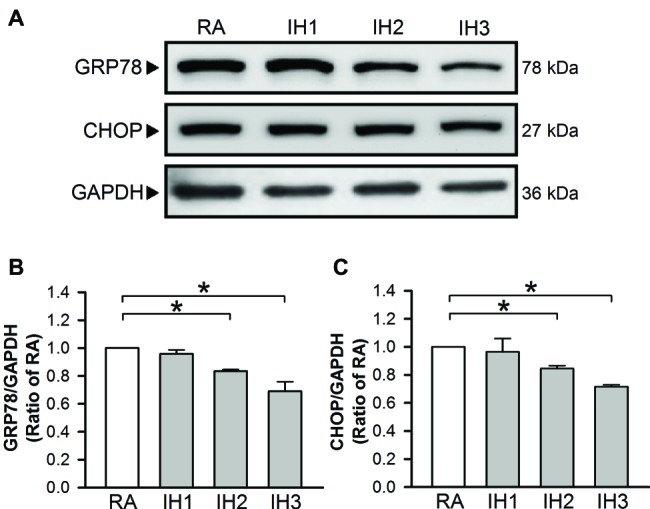
IH attenuates ER stress in H9c2 cells. Cells were exposed to IH for 1–3 days, and then harvested for detecting the GRP78 and CHOP protein levels. **(A)** A representative result of GRP78, CHOP, and GAPDH protein levels determined by Western blot analyses. Quantitative results shown in **(B)** for GRP78 protein levels and **(C)** for CHOP protein levels were adjusted with GAPDH protein level and expressed as ratio over the RA group. Values are means ± SEMs. (*n* = 4). *p < 0.05. IH, intermittent hypoxia; RA, room air.

To evaluate ER stress in cultured H9c2 cells, the levels of GRP78 and CHOP protein, indicators for ER stress were examined (Oslowski and Urano, 2011). Our results demonstrated that GRP78 and CHOP protein levels in cultured H9c2 cells were significantly decreased in the group of 2 or 3 days IH exposure as compared with the RA group (Figures 1A–C).

### Effects of Intermittent Hypoxia Exposure on the Thapsigargin-, Curcumin-, and H_2_O_2_-Increased Endoplasmic Reticulum Stress in Cultured H9c2 Cells

**Figure 2:**
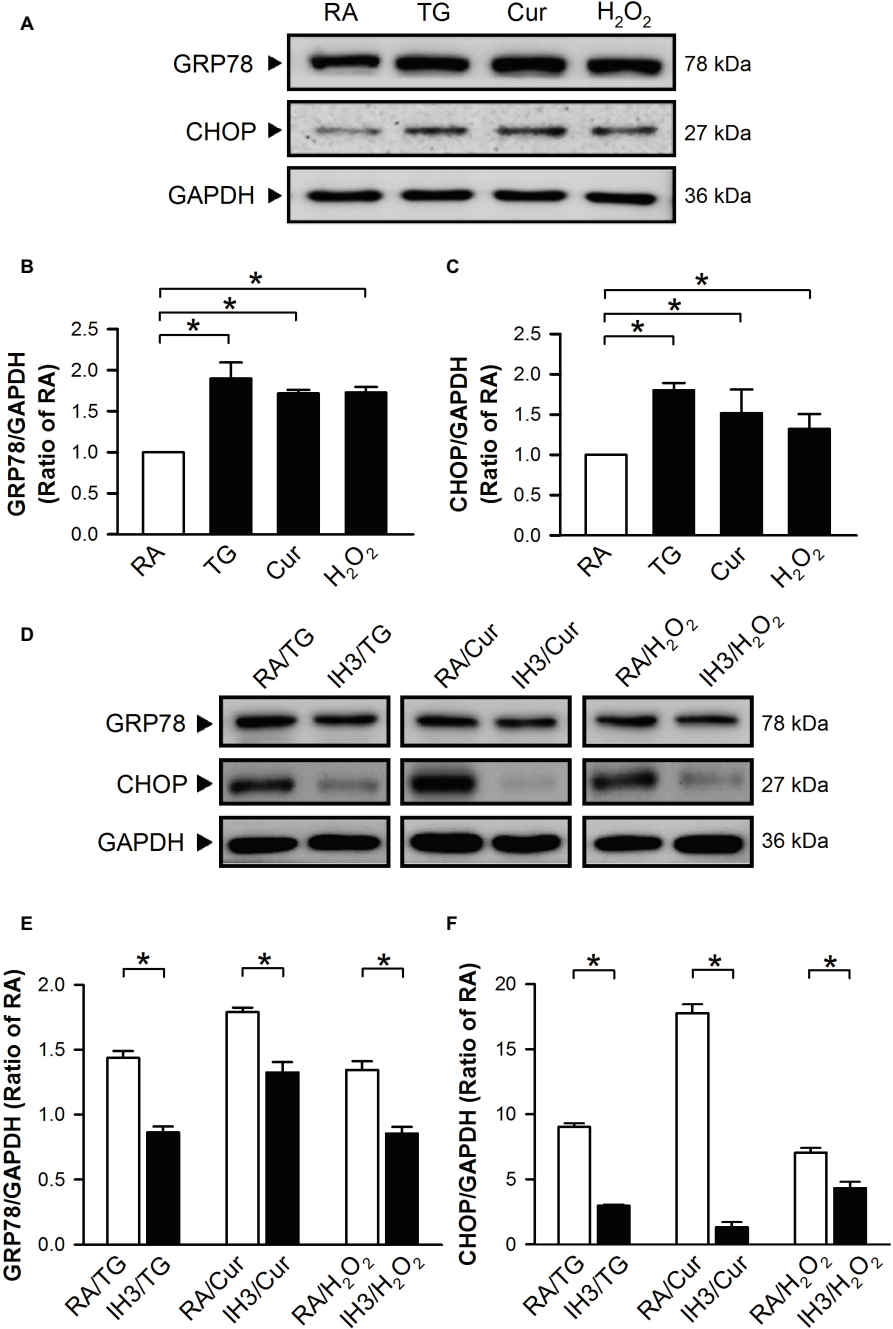
IH reduces the thapsigargin-, curcumin-, or H_2_O_2_-induced increases of ER stress in H9c2 cells. Cells were treated with TG (1 μM; 5 h), Cur (35 μM; 24 h), or H_2_O_2_ (100 μM; 24 h) **(A–C)** or with IH for 3 days followed by TG (5 h), Cur (24 h), or H_2_O_2_ (24 h) **(D–F)**, and then harvested for detecting the GRP78 and CHOP protein levels. **(A)** A representative result of GRP78, CHOP, and GAPDH protein levels determined by Western blot analyses. Quantitative results shown in **(B)** for GRP78 protein levels and **(C)** for CHOP protein levels were adjusted with GAPDH protein levels and expressed as ratio over the RA group. **(D)** A representative result of GRP78, CHOP, and GAPDH protein levels in RA-exposed or IH-exposed cells determined by Western blot analyses. Quantitative results shown in **(E)** for GRP78 protein levels and **(F)** for CHOP protein levels were adjusted with GAPDH protein level and expressed as ratio over the RA group. Values are means ± SEMs (*n* = 4). *p < 0.05. Cur, curcumin; ER, endoplasmic reticulum; IH, intermittent hypoxia; RA, room air; TG, thapsigargin.

To confirm the effect of IH on ER stress in H9c2 cells, thapsigargin (TG), a specific inhibitor of sarcoplasmic/endoplasmic Ca^2+^-ATPase (SERCA) (Oslowski and Urano, 2011; Sano and Reed, 2013), curcumin (Cur) (Wang et al., 2011; Moustapha et al., 2015), and H_2_O_2_ (Gardner et al., 1997; Yang et al., 2017) were used to increase ER stress in cultured H9c2 cells. Cells were treated with RA for 3 days followed by TG (1 μM) for 5 h, and Cur (35 μM) and H_2_O_2_ (100 μM) for additional 24 h, and then harvested for analyzing the levels of ER stress-related proteins, GRP78 and CHOP. Treatment with TG, Cur, or H_2_O_2_ significantly upregulated the levels of GRP78 and CHOP protein in cultured H9c2 cells as compared with the RA group (Figures 2A–C). However, on pre-treatment with IH for 3 days followed by TG (5 h), Cur (24 h) or H_2_O_2_ (24 h), the GRP78 and CHOP protein levels in the IH group were significantly reduced as compared with the RA group (Figures 2D–F).

### Association Between the Intermittent Hypoxia-Attenuated Endoplasmic Reticulum Stress and the Occurrence of Autophagy in Cultured H9c2 Cells

**Figure 3:**
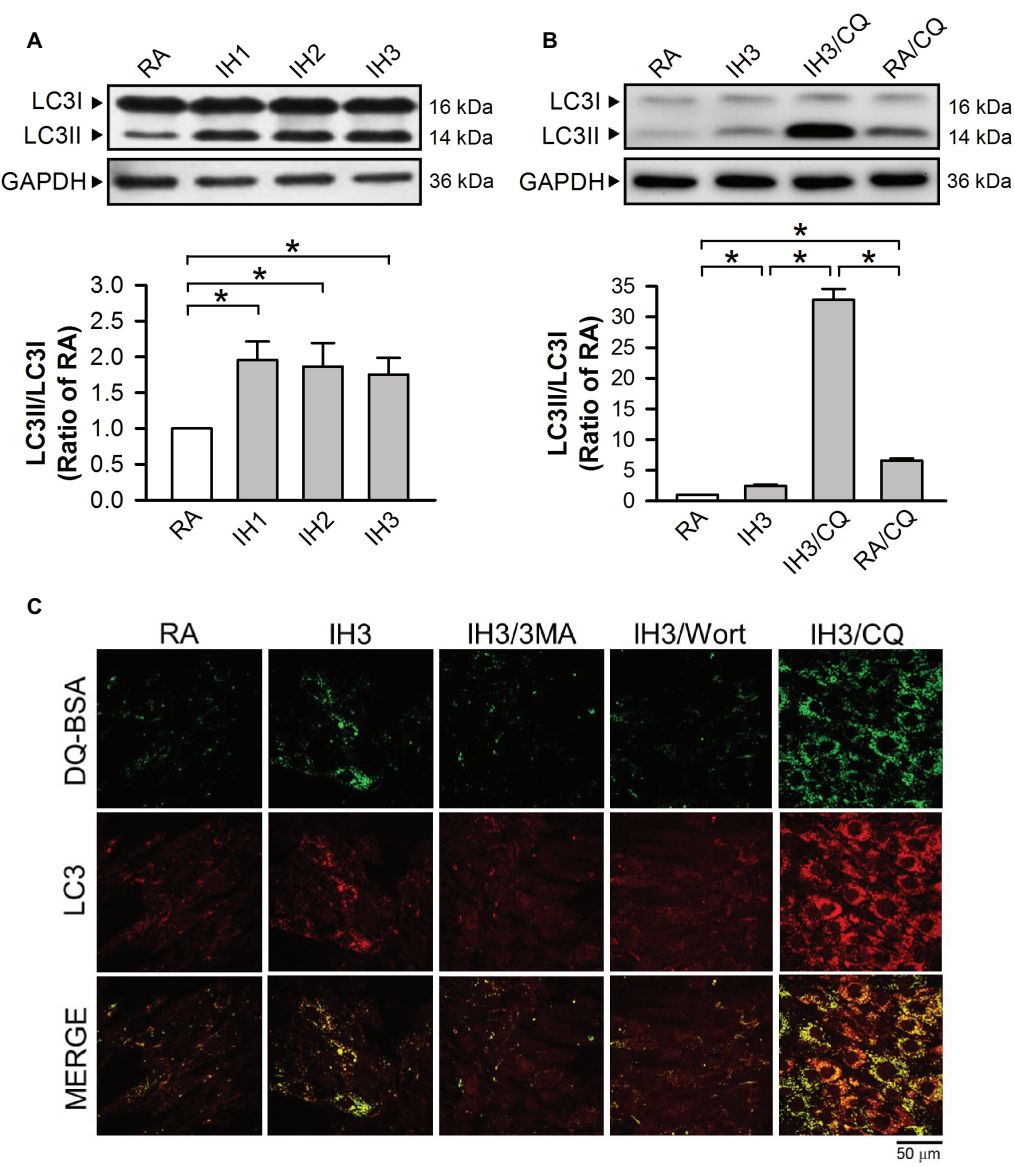
IH attenuates autophagy-associated ER stress in H9c2 cells. **(A)** Cells were treated with RA or IH for 1–3 days, and then harvested for detecting the LC3 protein levels. A representative result of LC3II and LC3I immunostaining in H9c2 cells determined by Western blot analyses. The protein levels of LC3II/LC3I were determined and expressed as ratio over the RA group. **(B)** Cells were treated with RA, IH for 3 days, IH for 3 days combined with chloroquine (5 μM), or RA combined with chloroquine (5 μM). A representative result of LC3II and LC3I immunostaining in H9c2 cells determined by Western blot analyses. Quantitative results of LC3I and LC3II protein levels were adjusted with the GAPDH protein level and the LC3II/LC3I value was expressed as ratio over the RA control group. **(C)** Cells were analyzed by confocal microscopy using DQ™-Green BSA. Autolysosomes were identified as LC3-positive (*red*)—green DQ-containing structures (*green*). The increased levels of autolysosomes were observed in IH-treated H9c2 cells. Cells were treated with RA or IH for 3 days followed by 3-methyladenine (50 μM), wortmannin (5 nM), or chloroquine (5 μM) for additional 24 h, and then analyzed by confocal microscopy using DQ™-Green BSA. The increased levels of autolysosomes were reduced by 3-methyladenine or wortmannin. Values are means ± SEMs. (*n* = 4). *p < 0.05. 3MA, 3-methyladenine; CQ, chloroquine; IH, intermittent hypoxia; RA, room air; Wort, wortmannin.

To examine the effect of IH on the occurrence of autophagy in cultured H9c2 cells, the ratios of LC3II to LC3I were adopted as an index of autophagy. Our results demonstrated that the ratios of LC3II to LC3I in cultured H9c2 cells were significantly increased by 1–3 days of IH exposure as compared to that exposed to RA (Figure 3A). To confirm that 3 days of IH could induce autophagy, the ratios of LC3II/LC3I in H9c2 cells treated with or without a lysosomal inhibitor chloroquine/bafilomycin A1 (CQ, 5 μM), which blocks autophagy flux at the final step of autophagy, were measured in both RA and IH groups. Our results showed that increased ratios of LC3II to LC3I were found in the cells treated with IH for 1–3 days and with CQ in both RA and IH3 conditions. Notably, CQ dramatically increased the ratio of LC3II to LC3I in the IH3 condition, suggesting that 3 days of IH can increase the levels of autophagy (Figure 3B). To further monitor the convergence of autophagosome and lysosome in the IH-induced autophagy, H9c2 cells were subjected to an evaluation of their ability to process DQ™-Green BSA (DQ-BSA); the green fluorescence is quenched unless DQ-BSA is hydrolyzed. As illustrated in Figure 3C, very little dequenched DQ-BSA was observed in RA-treated cells, whereas dequenched DQ-BSA was observed in IH-treated H9c2 cells. Notably, the dequenched DQ-BSA was colocalized with LC3-positive puncta in the IH-treated H9c2 cells, suggesting the occurrence of autophagy. To confirm that IH increased the levels of autophagy, 3-methyladenine (3MA, 50 μM), an autophagy early stage inhibitor, wortmannin (Wort, 5 nM), an inhibitor for LC3II conversion, or chloroquine (CQ, 5 μM) was applied to inhibit the occurrence of autophagy in the IH-treated H9c2 cells. Our results demonstrated that the effect of IH on the levels of dequenched DQ-BSA, which was colocalized with LC3-positive puncta, were abolished by co-treatment with 3MA or Wort. However, CQ, an autophagy inhibitor that significantly increases the amount of LC3B-II, enhanced the accumulation of the levels of dequenched DQ-BSA induced by IH, suggesting that IH induced autophagic flux (Figure 3C).

### Signaling Pathway of Intermittent Hypoxia-Attenuated Autophagy-Associated Endoplasmic Reticulum Stress in Cultured H9c2 Cells

**Figure 4:**
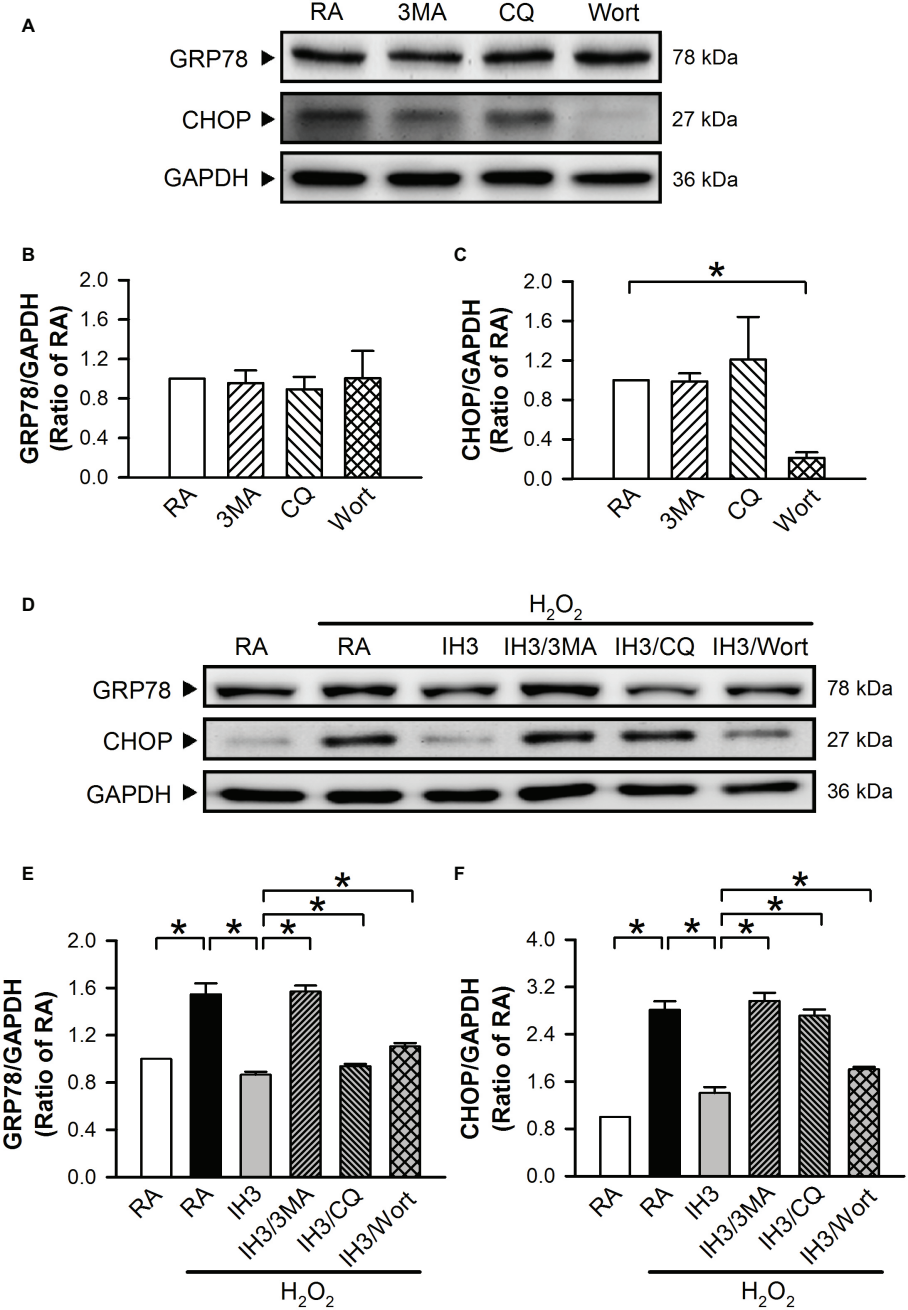
Involvement of autophagy in the IH-reduced ER stress in H9c2 cells. **(A)** A representative result of GRP78, CHOP, and GAPDH protein levels determined by Western blot analyses. Quantitative results of the protein levels of GRP78 **(B)** and CHOP **(C)** were adjusted with GAPDH protein level and expressed as ratio over the RA group. (*n* = 3). Cells were treated with RA, RA combined with 3MA, RA combined with CQ, or RA combined with Wort. **(D)** A representative result of GRP78, CHOP, and GAPDH protein levels determined by Western blot analyses. Quantitative results of the protein levels of GRP78 **(E)** and CHOP **(F)** were adjusted with GAPDH protein level and expressed as ratio over the RA control group. (*n* = 4). Cells were treated with RA, IH for 3 days, IH for 3 days combined with 3-methyladenine (50 μM), RA combined with chloroquine (5 μM), or wortmannin (5 nM). Values are means ± SEMs. *p < 0.05. 3MA, 3-methyladenine; CQ, chloroquine; IH, intermittent hypoxia; RA, room air; Wort, wortmannin.

**Figure 5:**
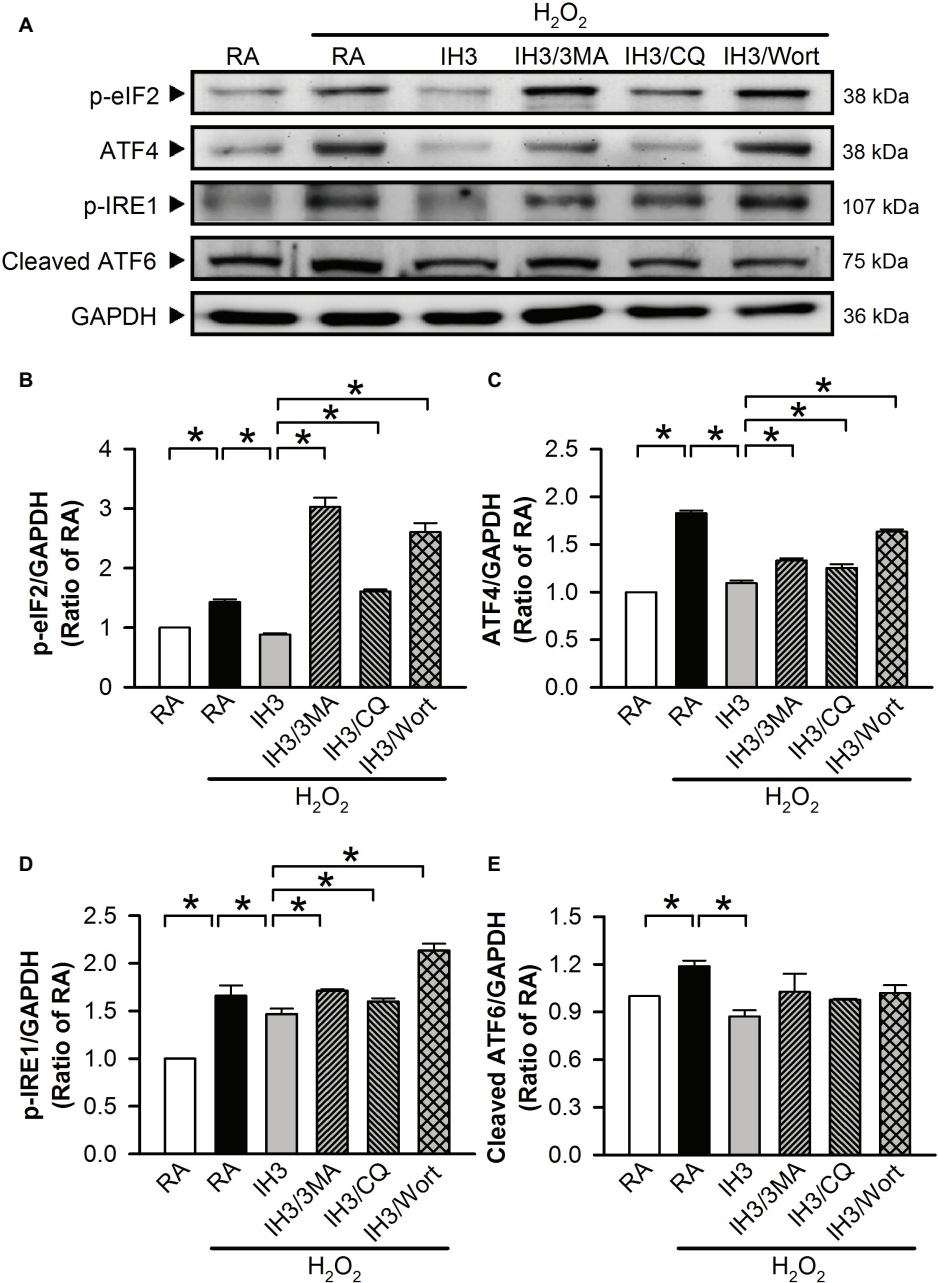
IH attenuates autophagy-associated ER stress through the p-elF2 and p-IRE1 signaling pathway in H9c2 cells. **(A)** Cells were treated with RA or IH for 3 days followed by H_2_O_2_ alone, H_2_O_2_ combined with 3-methyladenine (50 μM), H_2_O_2_ combined with chloroquine (5 μM), or H_2_O_2_ combined with wortmannin (5 nM) for additional 24 h, and then harvested for detecting the ER stress-related protein levels. **(A)** A representative result of p-eIF2, ATF4, p-IRE1, cleaved ATF6, and GAPDH protein levels determined by Western blot analyses. Quantitative results shown in **(B)** for p-eIF2 protein levels, **(C)** for ATF4 protein levels, **(D)** for p-IRE1 protein levels, and **(E)** for cleaved ATF6 protein levels were adjusted with GAPDH protein level and expressed as ratio over the RA group. Values are means ± SEMs. (*n* = 4). *p < 0.05. 3MA, 3-methyladenine; CQ, chloroquine; IH, intermittent hypoxia; RA, room air; Wort, wortmannin.

To examine whether autophagy plays a role in the IH-regulated ER stress, 3MA (50 μM), chloroquine (CQ, 5 μM), and Wort (5 nM) were applied to inhibit the occurrence of autophagy in cultured H9c2 cells. Initially, we detected the effects of 3MA, CQ, and Wort on ER stress-related proteins (GRP78 and CHOP) of cells in the RA group. The results showed that the levels of GRP78 between RA treated with and without the autophagy inhibitors were not significantly different (Figures 4A,B). The levels of CHOP between RA treated with or without the autophagy inhibitors were also not significantly different except for the Wort-treated group, in which it was significantly reduced (Figures 4A,C). These results suggested that the autophagy inhibitors used in this study had no significant effects on ER stress in RA, except the effect of Wort on CHOP. Next, we treated the cells with H_2_O_2_ to induce ER stress and then examined the effect of IH on the ER stress. As shown in Figures 4D–F, the levels of ER stress-related proteins, GRP78 and CHOP, were significantly increased by H_2_O_2_ treatment in the RA group, and this effect was abolished by IH treatment. However, the IH-mediated reduction of GRP78 and CHOP protein was significantly decreased by co-treatment with 3MA, CQ, or Wort (Figures 4D–F). These results suggest that IH attenuated ER stress through inducing autophagy. We further investigated which signaling pathways were involved in the IH-induced decreases of the autophagy-associated ER stress. Our data demonstrated that the levels of ER stress-related proteins (p-elF2, ATF4, p-IRE1, and cleaved ATF6) were significantly increased by H_2_O_2_ treatment, and these effects were significantly reduced by IH treatment (Figures 5A–E). However, the effect of IH on the levels of H_2_O_2_-increased ER stress-related proteins was significantly reduced by 3MA, CQ, or Wort (Figures 5A–E), except for the cleaved ATF6 (Figure 5E). These results suggest that inhibiting the elF2, ATF4, or IRE1 signaling pathway might contribute to the autophagy-reduced ER stress.

### Involvement of Autophagy in Intermittent Hypoxia-Attenuated Endoplasmic Reticulum Stress-Caused Cell Death in Cultured H9c2 Cells and Rat Primary Cultured Cardiomyocytes

**Figure 6:**
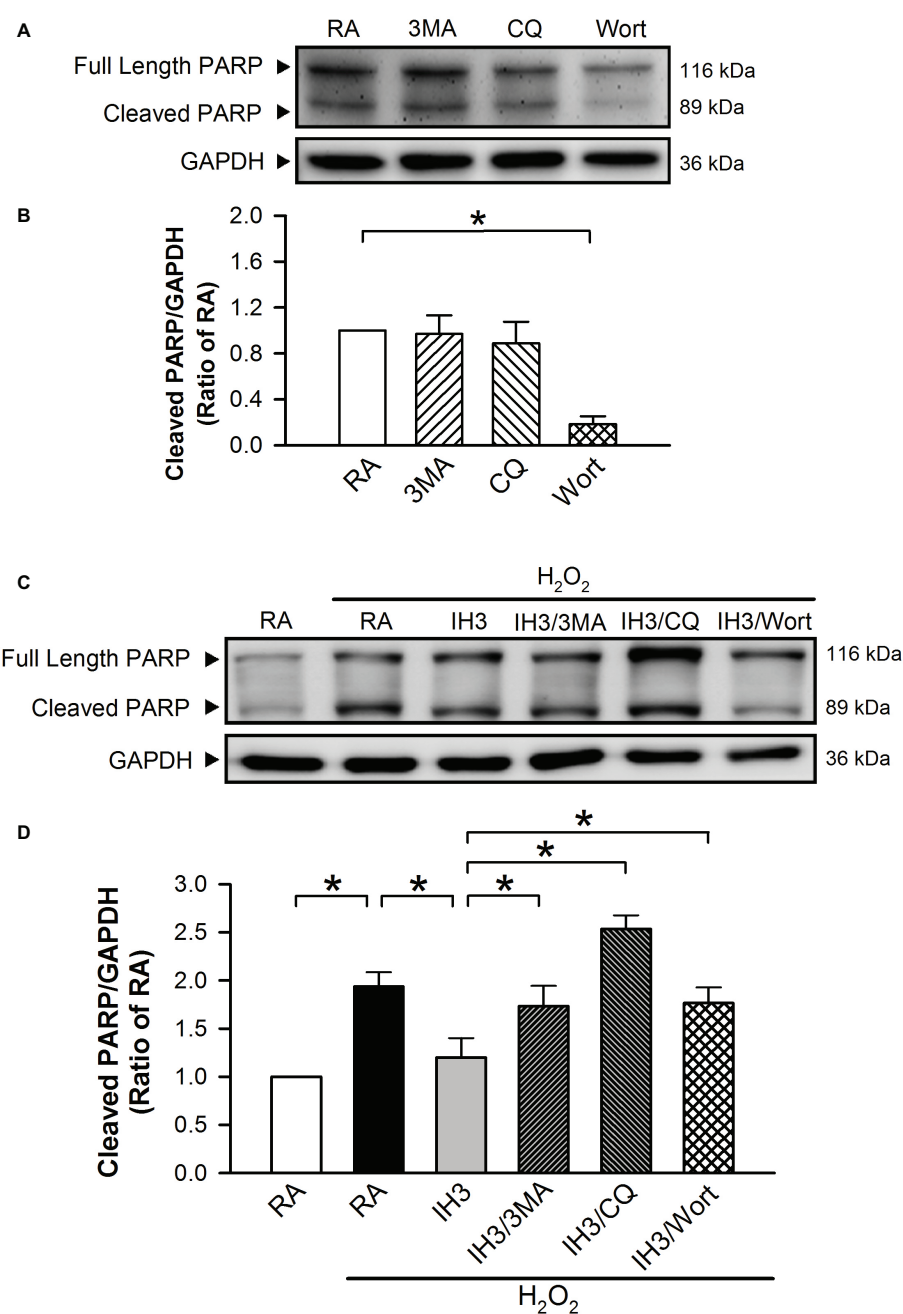
Involvement of autophagy in the IH-attenuated ER stress-induced cell death in H9c2 cells. Cells were treated with RA, RA combined with 3MA, RA combined with CQ, or RA combined with Wort. **(A)** A representative result of full length PARP, cleaved PARP, and GAPDH protein levels determined by Western blot analyses. Quantitative results of cleaved PARP protein levels were adjusted with GAPDH protein level and expressed as ratio over the RA group. (*n* = 4). **(B)** Cells were treated with RA or IH for 3 days followed by H_2_O_2_, H_2_O_2_ combined with 3-methyladenine (50 μM), H_2_O_2_ combined with chloroquine (5 μM), or H_2_O_2_ combined with wortmannin (5 nM) for additional 24 h, and then harvested for detecting the cleaved PARP and GAPDH protein levels. **(C)** A representative result of full length cleaved PARP, cleaved PARP, and GAPDH protein levels determined by Western blot analyses. **(D)** Quantitative results of cleaved PARP protein levels were adjusted with GAPDH protein level and expressed as ratio over the RA group. Values are means ± SEMs. (*n* = 3). *p < 0.05. 3MA, 3-methyladenine; CQ, chloroquine; IH, intermittent hypoxia; RA, room air; Wort, wortmannin.

**Figure 7:**
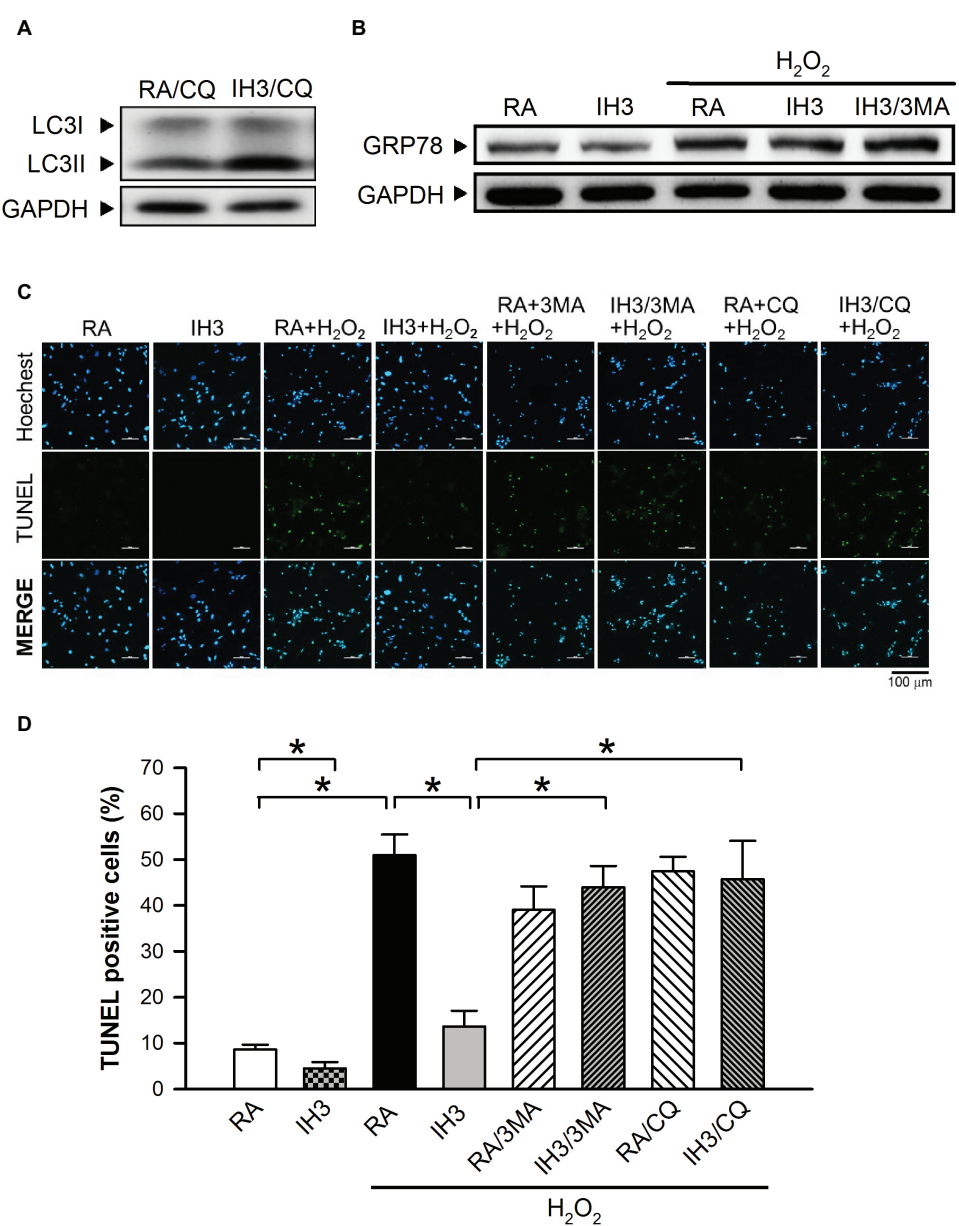
Involvement of autophagy in the IH-attenuated ER stress-induced cell death in cultured rat primary cardiomyocytes. **(A)** Cardiomyocytes were treated with RA or IH for 3 days followed by chloroquine (5 μM) for additional 24 h, and then harvested for LC3 protein level determination. A representative result of LC3 and GAPDH protein levels determined by Western blot analyses was shown. **(B)** Cardiomyocytes were treated with RA or IH for 3 days followed by H_2_O_2_, H_2_O_2_ combined with 3-methyladenine (50 μM) for additional 24 h, and then harvested for GRP78 protein levels determination. A representative result of GRP78 and GAPDH protein levels determined by Western blot analyses. **(C)** Cardiomyocytes were treated with RA or IH for 3 days followed by H_2_O_2_, H_2_O_2_ combined with 3-methyladenine (50 μM), H_2_O_2_ combined with chloroquine (5 μM), or H_2_O_2_ combined with wortmannin (5 nM) for additional 24 h, and then harvested for apoptotic cell detection. Cardiomyocytes were analyzed by confocal microscopy using TUNEL stain. Apoptotic cells were identified as TUNEL-positive (*green*). Nuclei were identified as Hoeshe-positive (*blue*). **(D)** Quantitative results of TUNEL-positive cells were expressed as ratio over the RA control group. Values are means ± SEMs. (*n* = 7). *p < 0.05. 3MA, 3-methyladenine; CQ, chloroquine; IH, intermittent hypoxia; RA, room air; Wort, wortmannin.

Accumulation of cleaved poly(ADP-ribose) polymerase-1 (PARP), an index of apoptosis, can trigger cells to undergo apoptosis. Initially, we detected the effects of 3MA, CQ, and Wort on the levels of cleaved PARP in H9c2 cells under RA conditions. The results showed that the levels of cleaved PARP were not significantly different between RA treated with and without the autophagy inhibitors, except that Wort treatment significantly reduced the level of cleaved PARA (Figures 6A,B). Moreover, our results showed that the cleaved PARP levels in the RA group were significantly increased by H_2_O_2_ treatment, and this effect was abolished by IH treatment (Figures 6C,D). However, the effect of IH on the H_2_O_2_-increased PARP cleavage in cultured H9c2 cells was significantly reduced by 3MA, CQ, or Wort treatment (Figures 6C,D). We also used the cultured rat primary cardiomyocytes to further confirm the role of autophagy in the IH-attenuated ER stress-induced apoptosis. Our results showed that CQ treatment induced a higher ratio of LC3II to LC3I in the IH condition than in the RA condition (Figure 7A). Consistent with the results done in H9c2 cells, a lower level of GRP78 protein was found in the IH group as compared to the RA group. Moreover, IH3 reduced the levels of GRP78 in the cells treated with or without H_2_O_2_. However, this effect mediated by IH3 was abolished by 3MA (Figure 7B). In addition, the apoptotic cells, detected by using TUNEL assay, were significantly increased by H_2_O_2_ treatment in the RA group, and this effect was abolished by IH treatment. However, the effect of IH on the H_2_O_2_-increased apoptotic cells was significantly reduced by 3MA, CQ, or Wort treatment in the rat primary cultured cardiomyocytes (Figures 7C,D). Taken together, our results suggested that the occurrence of autophagy might contribute to the reduction of the apoptosis resulting from the IH-attenuated ER stress in cultured cardiomyocytes.

### Involvement of Autophagy in the Intermittent Hypoxia-Attenuated Myocardial Endoplasmic Reticulum Stress in C57BL/6 Mice

**Figure 8:**
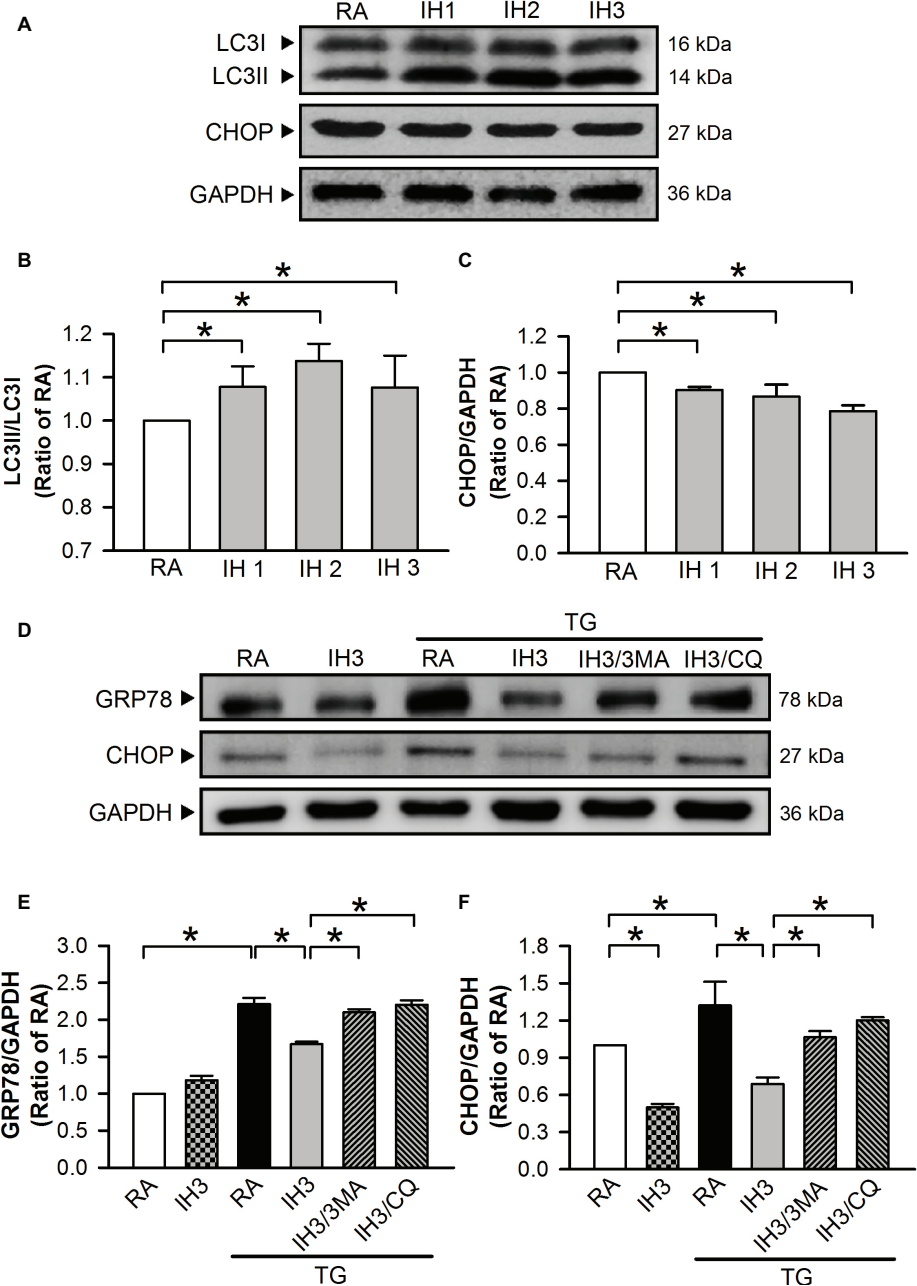
Involvement of autophagy in the IH-attenuated myocardial ER stress *in vivo*. C57BL/6 mice were exposed to RA or IH for 1, 2, or 3 days, or IH 3 days followed by thapsigargin (15 mg/kg/day), thapsigargin combined with 3-methyladenine (15 mg/kg/day), or thapsigargin combined with chloroquine (mg/kg/day), and then sacrificed for detecting the myocardial LC3, CHOP, GRP78, and GAPDH protein levels. **(A)** A representative result of LC3I, LC3II, CHOP, and GAPDH protein levels determined by Western blot analyses. Quantitative results of the protein levels of LC3II/LC3I **(B)** and CHOP/GAPDH **(C)** were expressed as ratio over the RA group. Values are means ± SEMs. (*n* = 3). **(D)** A representative result of GRP78, CHOP, and GAPDH protein levels determined by Western blot analyses. Quantitative results shown in **(E)** for the GRP78 protein levels and **(F)** for CHOP protein levels were adjusted with GAPDH protein level and expressed as ratio over the RA control group. Values are means ± SEMs. (*n* = 4). *p < 0.05. MA, 3-methyladenine; CQ, chloroquine; IH, intermittent hypoxia; RA, room air; TG, thapsigargin.

To examine the interplay of autophagy and ER stress in animal model, C57BL/6 mice were exposed to IH for 1, 2, or 3 days. Autophagy index was quantified by the conversion of LC3I to LC3II protein using Western blot analyses. The ratios of LC3II/LC3I were significantly increased (Figures 8A,B), while the levels of CHOP protein were decreased (Figures 8A,C) in the heart isolated from C57BL/6 mice treated with 1, 2, and 3 days of IH exposure. Since H_2_O_2_ is a strong oxidizing agent, which has been shown to cause systematic damage in animals, intraperitoneal injection of TG (1 mg/kg) instead of H_2_O_2_ was applied to induce myocardial ER stress. Our data demonstrated that myocardial levels of GRP78 and CHOP protein were significantly increased by TG injection, and this effect was significantly reduced by IH exposure. The effect of IH on the TG-increased GRP78 and CHOP protein levels was significantly decreased by i.p. injection with 3MA (15 mg/kg) or CQ (40 mg/kg) (Figures 8D–F). These results suggest that the occurrence of autophagy might contribute to the IH-attenuated ER stress in C57BL/6 mice.

### Effects of the Intermittent Hypoxia-Increased Myocardial Autophagy on the Endoplasmic Reticulum Stress-Caused Myocardial Apoptosis in C57BL/6 Mice

**Figure 9:**
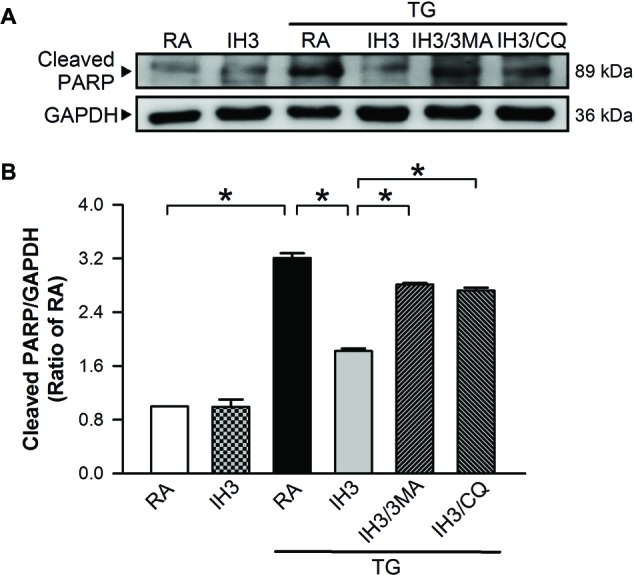
IH-induced myocardial autophagy contributes to the attenuation of ER stress-induced myocardial apoptosis *in vivo*. C57BL/6 mice were exposed to RA or IH for 3 days followed by thapsigargin (5 mg/kg/day) alone, thapsigargin combined with 3-methyladenine (15 mg/kg/day), or methyladenine combined with chloroquine (20 mg/kg/day), and then sacrificed for detecting the myocardial cleaved PARP and GAPDH protein levels. **(A)** A representative result of cleaved PARP and GAPDH protein levels determined by Western blot analyses. **(B)** Quantitative results of cleaved PARP protein levels were adjusted with GAPDH protein level and expressed as ratio over the RA control group. Values are means ± SEMs. (*n* = 3). *p < 0.05. MA, 3-methyladenine; CQ, chloroquine; IH, intermittent hypoxia; RA, room air; TG, thapsigargin.

Since mice are easier to perform gene manipulation on than rats, we chose C57BL/6 male mice as an animal model for the initial animal test of the effect of IH on ER stress and apoptosis. Injection of mice with TG increased the myocardial cleaved PARP levels, and this effect was significantly reduced by IH exposure (Figures 9A,B). Compared with TG-injected mice, however, the myocardial levels of cleaved PARP were significantly reduced in mice exposed to IH combined with TG injection (Figures 9A,B), suggesting that IH-induced myocardial autophagy might contribute to the reduction of the ER stress-caused myocardial apoptosis.

## Discussion

The major findings in this study revealed that the TG-, Cur-, or H_2_O_2_-increased ER stress in cardiomyocytes was abolished by IH exposure. Moreover, IH increased the levels of autophagy, and IH-decreased ER stress was abolished by autophagy inhibitors, 3MA, CQ, and Wort. These findings show for the first time that IH-increased autophagy levels might contribute to decreases of ER stress and apoptosis in cardiomyocytes *in vitro* and *in vivo*.

### Intermittent Hypoxia Attenuates Endoplasmic Reticulum Stress in H9c2 Cells

Previously, Belaidi et al. demonstrated that chronic IH (21–5% FiO_2_, 60 s/cycle, 8 h/day) for 14 days induces increases of ER stress markers, ER-Ca^2+^ content, HIF-1 activity, and infarct size in the hearts of C57B16J mice (Belaidi et al., 2016). IH induces cardiac pro-apoptotic ER stress characterized by upregulation of GRP78, phosphorylated protein kinase-like ER kinase, C/EBP homologous protein, and activating transcription factor 4 (Xu et al., 2015). The myocardial apoptosis caused by IH was confirmed by increased cleaved caspase-3 levels (Bourdier et al., 2016). Another chronic IH (CIH) study, in which rats were subjected to CIH conditions (5–6% O_2_) for 8 h/day for 5 weeks, demonstrated that the percentage of apoptotic cells in the CIH group was significantly higher than that in normal control group (2.95 vs. 0.42%) (Ding et al., 2014). Notably, in a 3 days to 3 weeks IH study [the IH paradigm consists of alteration cycles of 20.9% O_2_/8% O_2_ FIO_2_ (30 episodes/h) with 20 s at the nadir FIO_2_ during the 12-h light phase], IH exposure for 3 weeks significantly induced increases of cardiac ER stress and apoptosis evidenced by increases of GRP78, ATF6, and CHOP protein levels; activations of caspase-12 and capase-3; and decreases of the Bcl2/Bax ratio. However, exposure of mice to IH for 3 days did not induce significant changes in cardiac ER stress and apoptosis (Zhou et al., 2014). However, our present study showed that the ER stress evaluated by the levels of GRP78, CHOP, p-elF2, p-IRE1, and cleaved ATF6 proteins was significantly decreased in H9c2 cells exposed to IH for 2–3 days. The discrepancy between their and our results might be due to the difference of IH model between cell and animal, and the pattern of IH. To confirm our findings, thapsigargin (TG), a specific inhibitor of sarcoplasmic/endoplasmic Ca^2+^-ATPase (SERCA) (Oslowski and Urano, 2011; Sano and Reed, 2013), curcumin (Cur) (Wang et al., 2011; Moustapha et al., 2015), and H_2_O_2_ (Gardner et al., 1997; Yang et al., 2017) were used to induce increases of ER stress in cardiomyocytes as positive controls. However, the effects of curcumin on ER stress are controversial. It has been demonstrated that treatment with 20 μM of curcumin for 24 h could attenuate the ER stress in cardiomyocytes (Huang et al., 2015; Guan et al., 2019). In contrast, treatment with 20–25 μM of curcumin for 24 h induces ER stress in human liposarcoma cell line SW872 cells (Wang et al., 2011) and human hepatoma-derived Huh-7 cells (Moustapha et al., 2015). It seems that the effect of curcumin on ER stress is cell type dependent. In this study, the ER stress was significantly induced in H9c2 cardiomyocytes by treating curcumin (35 μM) for 24 h. Our results showed that TG-, Cur-, or H_2_O_2_-increased levels of ER stress were abolished by IH, suggesting that 3 days of IH could attenuate the ER stress in H9c2 cells.

### Intermittent Hypoxia Enhances Autophagy in H9c2 Cells

Autophagy mediates oxidative stress-elicited myocardial injury in response to ischemia/reperfusion (Mei et al., 2015). In animal models of cardiovascular disease, excessive or insufficient autophagy are each maladaptive, and a wide range of cardiovascular disorders are accompanied by robust increases in the autophagic activity (Morales et al., 2014). However, increased levels of exogenous ROS can significantly induce myocyte autophagy (Wang et al., 2018). In this study, we used the IH pattern (nadir 4% O_2_ to peak 20% O_2_ with 5% CO_2_, 1 cycle/h) suggested by a previous study showing that this IH pattern could induce an increase in ROS generation that did not cause death in cardiomyocyte (Chen et al., 2014). Thus, the IH pattern applied in this study is classified as an acute or mild paradigm that can induce increased levels of exogenous ROS to active autophagy evidenced by the increased levels of LC3II were found in H9c2 cells exposed to IH for 1–3 days.

### Intermittent Hypoxia-Attenuated Endoplasmic Reticulum Stress Is Associated With Autophagy in H9c2 Cells

It has been suggested that ATF4 plays an important role in regulating the ER stress-induced autophagy and provides a direct mechanistic link between the autophagic machinery and the UPR (Rzymski et al., 2010). Severe hypoxia (0.01% O_2_ for 24 h) induces ER stress and causes ATF4-dependent autophagy *via* LC3 as a survival mechanism (Rzymski et al., 2010). In contrast, under low-frequency, acute and mild hypoxia paradigm, the organism develops adaptive and protective mechanisms at the cellular, tissue, and organ levels to avoid cardiac, cerebral, and pulmonary damage (Sforza and Roche, 2016). The discrepancy between these two studies might be due to difference in the concentration of O_2_ and the pattern of IH (Rzymski et al., 2010). On the other hand, ROS derived from NADPH oxidases can activate the PERK/p-EIF2α/ATF4 pathway of the UPR by promoting phosphorylation of eIF2α and induction of ATF4 under ER stress conditions (Petry et al., 2019). Notably, a previous study suggested that NADPH oxidase is a major source of endogenous ROS that regulate myocyte autophagy in H9c2 cells (Wang et al., 2018). Thus, to examine whether autophagy is involved in the IH-attenuated ER stress, H9c2 cells were treated with H_2_O_2_ combined with autophagy inhibitors, 3MA, CQ, or Wort, under the IH condition. Our results showed that IH-induced attenuation of the H_2_O_2_-activated eIF2α/ATF4/CHOP was abolished by administration of autophagy inhibitors, 3MA, CQ, or Wort. On the other hand, molecules involved in the autophagy cascade from ULK1 to PI3P effectors, which are responsible for autophagosome nucleation, are localized to the ER, suggesting that ER is likely a candidate for the membrane source and the scaffold for autophagosome formation. Interestingly, the ER stress is also a trigger of autophagy. Under ER stress, the autophagy is induced through the IRE-1–JNK/p38 pathway and the ATF4 pathway in mammalian cells. However, pre-activation of autophagy by ischemic preconditioning can activate defense mechanisms and reduce excessive ER stress (Zhang et al., 2017). Recently, we reported that pretreatment with IH induced ROS generation, subsequently reducing severe oxidative stress (I/R, H_2_O_2_, DTDP, or NEM)-induced cardiomyocytes death (Lien et al., 2018). Therefore, our results suggested that pretreatment with IH may induce autophagy that contributed to attenuate the H_2_O_2_-induced ER stress in H9c2 cells.

### Intermittent Hypoxia-Attenuated Endoplasmic Reticulum Stress Is Associated With Apoptosis in H9c2 Cells and Cardiomyocytes

IH-increased autophagy may play a role in elimination of eIF2α, ATF4, and CHOP activation. However, Collett et al. showed that tunicamycin, a compound commonly used to induce ER stress, concentration dependently increases the levels of GRP78 and phosphorylated eIF2α but only at higher concentrations result in increases of CHOP level and PARP cleavage, suggesting that induction of severe ER stress might cause cell death (Collett et al., 2018). Moreover, increasing evidence demonstrated that UPR regulates both apoptotic and survival effectors, including CHOP and AATF. CHOP has been suggested to play a key role in the ER stress-caused β-cell death. Over-expression of CHOP reduces the Bcl-2 protein levels (Sano and Reed, 2013). Bcl-2 has been demonstrated to inhibit Bax translocation from the cytosol to the mitochondria. Since Bax participates in the ER stress-caused cell death, CHOP may induce apoptosis by enhancing pro-apoptotic components, such as Bax, and/or by suppressing anti-apoptotic genes, such as Bcl-2 (Ishigaki et al., 2010; Oslowski and Urano, 2010). In this study, we demonstrated that H_2_O_2_ increased the levels of GRP78 and CHOP protein, cleaved PARP in H9c2 cells, and the number of apoptotic cells in cultured rat primary cardiomyocytes. Moreover, IH attenuated the H_2_O_2_-increased cleaved PARP levels in H9c2 cells and the number of apoptotic cells in cultured rat primary cardiomyocytes, and this effect was abolished by autophagy inhibitors. These findings suggest that pretreatment with IH attenuated the ER stress-caused cell death through induction of autophagy in H9c2 cells and cultured rat primary cardiomyocytes.

We also used the IH animal model to examine whether 3 days of IH can increase autophagy, hence contributing to elimination of ER stress in the heart. In agreement with our *in vitro* studies conducted in rat primary cultured cardiomyocytes, our *in vivo* studies showed that increased levels of LC3II and decreased levels of GRP78 and CHOP were found in 1–3 days of IH exposure in mice. Moreover, IH could attenuate the levels of myocardial ER stress and cleaved PARP induced by TG, and these effects were abolished by autophagy inhibitors.

### Clinic Application and Significance

For now, physicians are still struggling to find effective reperfusion therapies to reduce the myocardial reperfusion injury. Recently, there have been a number of scientific advancements in identifying and understanding the factors involved in cell death caused by reperfusion and, based on these studies, potentially useful therapeutic new targets have been identified for preventing the myocardial reperfusion injury (Ruiz-Meana and Garcia-Dorado, 2009). Notably, it has been suggested that IH is also a mechanical therapeutic intervention. Hayashi et al. reported that 10 days of IH enhances left ventricle systolic function and right ventricle pressure-loading (Hayashi et al., 2011). However, cardiomyocytes apoptosis and LV systolic dysfunction were found in a longer duration of IH (8 weeks) (Chen et al., 2010). Thus, short-term IH might cause autophagy, hence promoting cardiac adaptations to maintain or even improve systolic function in response to hemodynamic and hypoxic stress (Maeda et al., 2013). This study demonstrated that IH-induced myocardial autophagy attenuated the ER stress and apoptosis, suggesting that IH can be applied as an adjunctive cardioprotection therapy to enhance the efficacy of thrombolysis and percutaneous coronary intervention, resulting in the attenuation of myocardial reperfusion injury.

### Limitations

Although the results from the present study suggest that IH-induced myocardial autophagy might contribute to the attenuation of ER stress and apoptosis in cultured H9c2 cells and rat primary cardiomyocytes and in the heart tissue of C57BL/6 mice, some issues remain to be addressed. First, the IH pattern conducted in the present study (75 s/cycle, 4% O_2_ to peak 20% O_2_ with 5% CO_2_) differs from other IH-related disease models, such as periodic types of breathing and chronic lung disease. Thus, the IH pattern, which caused myocardial autophagy or ER stress leading to myocardial apoptosis, might differ from those IH-related disease models, such as the inflammation responses-caused cardiac remodeling and dysfunction (Zhou et al., 2014). Second, the association between ROS and accumulation of unfolded proteins in the ER was not studied in the present study. The relationship among ROS, autophagy, and ER stress deserves further investigation. Third, although our data suggest that IH-increased autophagy might protect cell from death by attenuation of ER stress and these findings might apply to study the cardioprotective effect against IR injury, the effects of IH on the whole body, such as hormone regulation and oxidative stress damage, still need to be further studied.

## Conclusion

Our data suggest that IH could increase myocardial autophagy as an adaptive response to prevent the ER stress and apoptosis *in vitro* and *in vivo*.

## Data Availability

The raw data supporting the conclusions of this manuscript will be made available by the authors, without undue reservation, to any qualified researcher.

## Ethics Statement

All experimental and surgical procedures were performed following the recommended procedures approved by the Institutional Animal Care and Use Committee of Tzu Chi University (PPL number: 107104), and conform to the guidelines from Directive 2010/63/EU of the European Parliament on the protection of animals used for scientific purposes or the NIH guidelines.

## Author Contributions

K-TY and T-IC conceived and designed the experiments. J-CC, W-FH, J-HL, and P-CT performed the experiment. K-TY, H-RC, K-RS, and T-IC contributed reagents, materials, and analysis tools. J-CC, W-FH, W-SL, J-HL, and P-CT analyzed the data. W-SL, T-IC, and K-TY wrote the paper. All authors read and approved the final manuscript.

### Conflict of Interest Statement

The authors declare that the research was conducted in the absence of any commercial or financial relationships that could be construed as a potential conflict of interest.
